# The co-production of what? Knowledge, values, and social relations in health care

**DOI:** 10.1371/journal.pbio.2001403

**Published:** 2017-05-03

**Authors:** Angela Filipe, Alicia Renedo, Cicely Marston

**Affiliations:** London School of Hygiene and Tropical Medicine, London, United Kingdom; City, University of London, United Kingdom

## Abstract

“Co-production” is becoming an increasingly popular term in policymaking, governance, and research. While the shift from engagement and involvement to co-production in health care holds the promise of revolutionising health services and research, it is not always evident what counts as co-production: what is being produced, under what circumstances, and with what implications for participants. We discuss these questions and propose that co-production can be understood as an exploratory space and a generative process that leads to different, and sometimes unexpected, forms of knowledge, values, and social relations. By opening up this discussion, we hope to stimulate future debates on co-production as well as draw out ways of thinking differently about collaboration and participation in health care and research.

Part of the title of this article is inspired by the book “The Social Construction of What?” by Ian Hacking (Cambridge, MA: Harvard University Press; 2000).

This Perspective is part of the Public Engagement in Science series.

“Co-production” was originally coined in the late 1970s, by economist Elinor Ostrom, to designate a process in which contributions from “individuals who are not “in” the same organization are transformed into goods and services” [1], including the assessment, management, and delivery of a public service by “users” and “providers.” This idea was further developed by academics and activists such as Edgar Cahn to include principles of social justice [2]. Today, co-production is a hot topic in policymaking, governance, and research, as well as in a range of fields from museums to climate change [3,4]. In health, and alongside user and community participation, co-production is described as a way of working together to improve health and of creating user-led, people-centred health care services [5]. In the United Kingdom, “co-production” has become a mainstream term in government and public policy discourse [6,7] and described in the media as the most radical of all approaches to National Health Service (NHS) reform [8].

Despite an apparent consensus around the potential of co-production, it is not always clear what counts as or what is meant by “co-production,” what it entails in practice, or what it is that is being co-produced. This is partly because different fields (e.g., public management, voluntary sector, and health services) embrace distinct visions of what the process is and aims to achieve. A recent report from the New Economics Foundation describes co-production as a value-driven approach that blurs barriers between the state, services, and citizens; involves relationships of reciprocity and mutuality; and applies an assets-based (as opposed to a deficit) model of service users [9]. The other reason there’s so much diversity and variation within co-production is that its meaning and scope change according to what is being produced, how, by whom, and to which purpose. In health care, for example, processes of co-production can take many forms, including the co-design, co-evaluation, and co-implementation of services and service improvements by patients, clinicians, carers, and managers with and without a research component [10,11]. Added together, these elements suggest that there are several idioms [12] and versions of co-production [13].

Yet, there is a common denominator amongst all the different approaches to and forms of co-production: the relationships that allow co-production to happen [10] and the new forms of knowledge, values, and social relations that emerge out of co-productive processes. In particular, we emphasise the complex, dynamic nature of these processes, as they not only take the form of interactions between individuals and services, but also involve interactions between different rationales for participation and policy agendas, between different modes of knowledge production (e.g., knowledge based on biomedical evidence, clinical practice, or experience of illness), and between different kinds of value (e.g., economic value and values of equity and social justice). As proposed by Jasanoff in the field of science and technology studies (STS), the concept of co-production may be used to describe how the “domains of nature, facts, objectivity, reason, and policy [cannot be separated] from those of culture, values, subjectivity, emotion, and politics” [12]. Similarly, the notion of co-production of value and services in health care cannot be dissociated from the values and implications of co-producing knowledge or the meanings of participation as a social and political process.

Today’s world is increasingly driven by knowledge economies and managerial demands in which certain types of knowledge and productivity rank above others as sources of evidence and value (e.g., metrics, evidence-based medicine). Asking what is being co-produced and how raises a set of wider questions about the rationale and scope of citizen participation and patient involvement relating to the distribution of expertise, power, and resources in health care and research and the social, material, and experimental dimensions of working together and across communities, disciplines, and/or organisations. In this short article, we explore these questions by drawing on our research on involving patients and members of the public in health care and service improvement in the UK.

It is essential to focus on the challenges and stakes of doing co-production in this context, as well as examining what is being produced and with what implications for participants. To this end, we offer a social science perspective that considers different understandings of co-production [14] and draws on research from the fields of health, education, participation, and STS. Thus, we contribute to a more ecological understanding of co-production than the one currently offered by some of the management literature, which tends to focus on co-production barriers, drivers, and motives while neglecting some of its experimental, relational, and normative dimensions. We propose that co-production should be viewed as an exploratory space that brings together different values and social relations and a generative process that produces new interactions and forms of knowledge and that can lead in turn to meaningful ways of shaping and taking part in health care.

## From being involved to co-producing health care

Co-production is seen in current policy agendas both as the next logical step to patient involvement and public engagement (PPI/E) and as a way of incorporating people’s expertise into health and social services and research ethics in more substantive and meaningful ways [15,16]. One of its distinctive features involves bringing citizens, service users, and communities into the decision-making process [14] by reducing social distance and knowledge and power imbalances between different participants and erasing artificial distinctions between “recipients” and “providers” of services [17]. While the shift from engagement and involvement in health care to the co-production of services and research holds a revolutionary promise, processes of co-production may have to build on and thus become entangled in existing involvement frameworks and practices [18].

In our ethnographic study of patient involvement and quality improvement initiatives in the NHS and in the National Institute for Health Research (NIHR) Collaboration for Leadership in Applied Health Research and Care program (CLAHRC) for Northwest London [19,20], we explored how different modes of knowledge are shared, produced, and translated into practice through clinical–researcher and patient–professional collaborations and participatory processes. Through observations and interviews, we found that patients, carers, PPI managers, and clinicians share the belief that co-production is of economic value and in the public interest. A common aim in this context is improving service availability, continuity, and quality [10]. Yet, they also put forward different arguments about why and how that might be the case; arguments that carry distinctive and sometimes conflicting meanings and values. These include: the **rights-based argument**, which holds that people have a right to participate in decisions that directly affect them; a **managerial logic**, in which co-production is a strategy for improving cost-effectiveness and adding value to services and projects; and an **experience-based argument**, in which narratives and experiences of illness, services, and treatment contribute to service improvement, knowledge, and health research [21].

In what we summarise as the idea of “co-production of value,” some health care professionals in the program defined co-production in terms of improving cost-effectiveness, consumer satisfaction, and greater patient–professional cooperation. They referred, for example, to patient involvement and experience as “adding value” to services and projects. Other participants saw co-production as a way of moving beyond token involvement and consultation towards more equitable power relations and more meaningful forms of participation and knowledge production through genuine collaboration—what could be called the “rights and values of co-production.” These views reflect not only diversity in and overlaps between participation and co-production but also within quality improvement, in which the fields of public engagement and new public management, health economics, and improvement and implementation sciences intersect and sometimes collide.

While this picture of conflation (and sometimes friction) may generate ambivalence and even political tensions among participants and stakeholders, it also provides the backdrop for some of the challenges and stakes inherent in co-production in this context. These include conflicting ideas about what is meant by “adding value” and the “patient perspective” [22] and what counts as labour, productivity, and value in health care and research.

## The challenges and stakes of doing co-production

### Putting “co-production” into practice

As a policy term, co-production benefits from retaining a degree of ambiguity. Although the lack of a strict definition can complicate efforts to get collaborations off the ground, it also allows more flexibility by expanding [23] rather than constraining what they might entail. This challenge is not simply a problem of translational “gaps” between policy and practice: it is a matter of organisational dispositions and positions, of personal attributions, and of conflicting assumptions about what co-production is and does in the context of health care. For instance, while some of the people we interviewed saw in co-production an opportunity to “revolutionise” health services, others feared it could turn into “a bit of a fad” if used simply as a way to rebrand PPI/E that risks subsuming the right to participation and the political nature of involvement to an economic discourse of production by partnership. This means that the process of co-production must take into account the participants’ understandings of participation and co-production, salient differences between them (e.g., identity, mobility, forms of communication), and power dynamics that may be reconfigured through the process of co-producing services and research. Such a process involves dialogue and recognition of each other’s capabilities and knowledge [24], while also allowing critical inquiry and the confrontation of ideas [7].

### Beyond economic value and “good” governance

In its original economics context, the term co-production offers an alternative view of service and value creation [17]. In health care, this notion also challenges how resources are allocated, how they are distributed among participants, and who takes part. A common question is whether and how health service users should be compensated for their time, which involves sharing their knowledge, for example, regarding experiences of care and illness or contributing ideas and technical expertise. In our experience, some users who are called to participate and co-produce say they do not need or want financial compensation; others would welcome it but for some compensation jeopardize their social security benefits. An uncritical application of the principle of seeing patients and carers as assets and equal contributors (as opposed to passive recipients of care and services) may miss out on substantive imbalances between them and paid professionals, while a simplistic notion of “co-everything” without adequate financial resources and parameters (e.g., timely refund of expenses) risks turning users into precarious participant-labourers. The programme we observed attempted to avoid this by using available resources to improve participant equity (e.g., paying for first class disability train tickets for those who needed them) and by funding user-led projects [25]. Commonly held notions of participation and co-production as voluntary acts that are unpaid (or paid below market-value) [13] should be reassessed in favour of a more substantive notion of co-production as a form of collaboration toward social justice, inclusion, and economic solidarity.

### Experimental and relational dimensions of co-production

Processes of co-production may yield unexpected insights into gaps in medical knowledge, healthcare needs, and/or service improvements. For example, a research theme around “breathlessness” (as opposed to the specific diagnostic categories of asthma and chronic obstructive pulmonary disease) was adopted by the program after being proposed by a service user who drew on knowledge grounded in experiences of illness and services. Another example was the co-design of tools that may improve and help integrate physical health care into mental health services by users and health care professionals. At the same time, processes of co-production may help legitimise intangible forms of labour (e.g., personal and professional investment, time commitment) that tend to be under-valued in health care; they also draw attention to material conditions for and immaterial challenges of meaningful participation and co-production. These include but are not limited to: devising new spaces for involvement [19] and for sharing knowledge and learning as well as sustaining these spaces and processes over the long term. These are material, relational, and temporal elements that tend to be neglected in the policy literature and in conventional framings of engagement based in meetings that are characterised by predefined agendas, minute-taking, and fast-paced conversations. Co-production calls, instead, for a slower pace of sharing and producing knowledge [26] and for open-ended, experimental [27,28] forms of learning, critical thinking [29], and working together in health care and research.

## Co-production as an exploratory space and generative process

One way of going about the co-production of health care more meaningfully is to look at it as a dynamic, experimental, and reflective process sustained by different forms of engagement, interactions, and social relations and that may generate, in turn, **new forms of care other than health care** (e.g., inclusive relationships, solidarity), **values beyond economic value** (e.g., equity, justice), and **new insights and research practices** that are relevant to different disciplines and practices (e.g., community participation, patient advocacy, collaborative research).

Given the predominance of cost-effectiveness and data-intensive work in health care and research, this substantive approach to co-production may be a hard sell to researchers, funders, and policymakers worried about limited resources and timeframes. In our view, emerging forms and processes of co-production can reveal and help legitimise possibilities that were unanticipated or even unthinkable before they began. Such possibilities may include new research insights, redistribution of resources, recognition of intangible forms of labour in health care, and the creation and renegotiation of social relations and participatory processes over the long term (that is, maintaining communities rather than simply delivering services).

Co-production is an exploratory social space that may challenge conventional framings of engagement, involvement, and voluntarism as well as commonly held notions of authority, capability, credibility, and productivity in contemporary health care and research. For this reason, co-productive experiments are best seen as generative processes that are less about delivering predictable impacts and outputs and more about developing new communities, interactions, practices, and different modes of knowledge and value production. We hope this experimental and ecological perspective on co-production may stimulate future debates and bring out different ways of shaping and participating in health care.

## Ethics statement

Both NHS and the London School of Hygiene and Tropical Medicine Research Ethics Committees approved the study, and we obtained written consent from interviewees.

## Abbreviations

NHS: National Health Service
PPI/E: patient involvement and public engagement
NIHR CLAHRC: National Institute of Health Research Collaboration for Leadership in Applied Health Research and Care
STS: science and technology studies
